# Anti-IL-6 Receptor Antibody Inhibits Spontaneous Pain at the Pre-onset of Experimental Autoimmune Encephalomyelitis in Mice

**DOI:** 10.3389/fneur.2019.00341

**Published:** 2019-04-09

**Authors:** Kenichi Serizawa, Haruna Tomizawa-Shinohara, Hideyuki Yasuno, Kenji Yogo, Yoshihiro Matsumoto

**Affiliations:** ^1^Product Research Department, Chugai Pharmaceutical Co., Ltd, Shizuoka, Japan; ^2^Product Research Department, Chugai Pharmaceutical Co., Ltd, Kanagawa, Japan

**Keywords:** IL-6, anti-IL-6 receptor antibody, experimental autoimmune encephalomyelitis, neuropathic pain, spontaneous pain, neuromyelitis optica, multiple sclerosis

## Abstract

Chronic pain is a significant symptom in patients with autoimmune encephalomyelitis, such as multiple sclerosis and neuromyelitis optica. The most commonly used animal model of these diseases is experimental autoimmune encephalomyelitis (EAE). We previously reported that evoked pain, such as mechanical allodynia, was improved by an anti-IL-6 receptor antibody in EAE mice. However, few reports have evaluated spontaneous pain in EAE mice. Here, we assessed spontaneous pain in EAE mice by utilizing the Mouse Grimace Scale (MGS, a standardized murine facial expression-based coding system) and evaluated the influence of an anti-IL-6 receptor antibody (MR16-1). EAE was induced in female C57BL/6J mice by subcutaneous immunization with myelin oligodendrocyte glycoprotein 35–55 emulsified in adjuvant and administration of pertussis toxin. Mice were placed individually in cubicles and filmed for about 10 min. Ten clear head shots per mouse from the video recording were given a score of 0, 1, or 2 for each of three facial action units: orbital tightening, nose bulge, and ear position. Clinical symptoms of EAE were also scored. Measurement of 5-HT in the spinal cord and functional imaging of the periaqueductal gray (PAG) were also performed. Compared with control mice, MGS score was significantly higher in EAE mice. MR16-1 prevented this increase, especially in pre-onset EAE mice. Promotion of spinal 5-HT turnover and reduction of PAG activity were observed in pre-onset EAE mice. These results suggest that MR16-1 prevented spontaneous pain developed before EAE onset.

## Introduction

Chronic pain is a major symptom associated with demyelinating autoimmune diseases of the central nervous system (CNS), such as multiple sclerosis (MS) and neuromyelitis optica (NMO). It has been reported that ~50–80% of patients with MS or NMO feel severe pain (1–3), and neuropathic pain is the most prevalent and most difficult to treat in these patients (4, 5).

Proinflammatory cytokines such as interleukin-6 (IL-6) can be important factors in neuropathic pain (6). Intrathecal injection of IL-6 induced allodynia (7), and IL-6 knockout mice showed milder pain symptoms (8, 9). Anti-IL-6 antibodies attenuated neuropathic pain in a peripheral nerve injury model (10) and anti- IL-6 receptor antibodies attenuated neuropathic pain in a spinal cord injury model (11). We also previously reported that a rat anti-mouse IL-6 receptor monoclonal antibody (MR16-1) improved mechanical allodynia in experimental autoimmune encephalomyelitis (EAE) mice (12), which is a well-established animal model of CNS autoimmune disease, and causes widespread CNS inflammation, demyelination, and locomotor impairments (13). Recently, some studies have demonstrated that EAE mice show neuropathic pain behavior (14–16).

Patients with neuropathic pain have complicated symptoms associated with both evoked and spontaneous pain (17), and mainly suffer from spontaneous pain (18, 19). The mechanisms of spontaneous pain are partly distinct from those of evoked pain, and therapeutic responses to evoked and spontaneous pain are different (20). Therefore, it is important to examine both spontaneous and evoked pain. Nevertheless, pain studies in EAE mice have mainly focused on evoked pain, and the effects of anti-IL-6 receptor antibodies on spontaneous pain in EAE mice have not been examined.

In the present study, therefore, we evaluated the analgesic effect of an anti-IL-6 receptor monoclonal antibody against spontaneous pain in EAE mice.

## Materials and Methods

### Animals

Totally 80 female C57BL/6J mice (7 weeks old; Charles River Laboratories Japan, Inc., Kanagawa, Japan) were used. If clinical score 4 or more was observed before the final assay point, mice were euthanized immediately as ethical endpoint. In functional imaging, pre-onset EAE mice were used for analysis. All mice were fed ordinary laboratory chow and allowed free access to water under a constant light and dark cycle of 12 h. All experimental protocols were approved by the Animal Care Committee of the institution (approved No. 18-011, 18-335) and conformed to the *Guide for the Care and Use of Laboratory Animals* published by the US National Institutes of Health.

### Induction of EAE

EAE was induced by subcutaneous immunization with 50 μg of the myelin oligodendrocyte glycoprotein 35–55 peptide (MOG_35−55_; Peptide International, Louisville, KY, USA) emulsified in complete Freund's adjuvant (Difco Laboratories, Detroit, MI, USA) supplemented with *Mycobacterium tuberculosis* extract H37Ra (Difco Laboratories) on Day 0. In addition, mice received 250 ng pertussis toxin (List Biological Laboratories, Campbell, CA, USA) intravenously on Day 0 and intraperitoneally on Day 2. Control mice were treated with complete Freund's adjuvant and saline alone. EAE mice were divided into two groups based on the body weight, and treated vehicle or MR16-1.

### Drugs

MR16-1 was prepared using a hybridoma established in our laboratory (21). EAE mice were intraperitoneally administered MR16-1 (8 mg/mouse) or vehicle on Day 12 after MOG immunization. The dosage of MR16-1 was determined by reference to the previous reports, and this timing of administration is known not to suppress clinical symptoms in EAE mice (12, 22).

### Clinical Score Assessment

Clinical symptoms of EAE were scored according to the following scale: 0, no disease; 1, limp tail; 2, hind limb weakness; 3, hind limb paresis; 4, hind limb paralysis; 5, hind limb and fore limb paralysis; 6, moribundity and death.

### Measurement of Mouse Grimace Scale

To assess spontaneous pain, we utilized the mouse grimace scale (MGS), a standardized murine facial expression-based coding system (23). Briefly, mice were placed individually in cubicles (9 × 5 × 5 cm) with transparent front and rear walls and opaque side walls. Mice were then filmed for about 10 min with two video cameras (one at each end). Ten clear head shots per mouse were taken from the video recordings, and each shot was given a score of 0, 1, or 2 for each of three facial action units: orbital tightening, nose bulge, and ear position. In scoring of the mouse photos, we encoded each animal's ID to blind the treatment group.

### LC-MS/MS Analysis

Mice were anesthetized with isoflurane, and transcardial perfusion was carried out with 20 mL of cold phosphate-buffered saline (PBS). Next, the L3–L5 segment of the lumbar spinal cord was removed, weighed, and homogenized using a Biomasher II (Nippi, Tokyo, Japan) with 100 μL ice cold 0.1% formic acid (FA). The homogenate was mixed and centrifuged at 15,000 rpm for 15 min at 4°C. The supernatant was transferred and added acetonitrile with 0.1% FA containing isoproterenol. The mixture was evaporated and reconstituted in 0.1% FA. A 10 μL sample was injected into the LC-MS system for analysis. A quadrupole mass spectrometer QTRAP 5500 (AB SCIEX, Framingham, MA, USA) was used for identification and quantification of serotonin (5-HT) and 5-HIAA, which is the major metabolite of 5-HT.

### Functional Imaging

Functional MRI (fMRI) analysis was performed by BioView Inc. (Tokyo, Japan) in EAE mice on Day 19, 20, or 21 after immunization. Briefly, structural T1-weighted and fMRI data were collected using a Varian 4.7T scanner (Unity INOVA, Varian Associates, Palo Alto, CA, USA). Before each scanning session, an exogenous contrast agent, USPIO (CL-30Q02-7; BioPAL Inc., Worcester, MA, USA), was injected into the caudal vein to optimize the localization of fMRI signals (24). GLM-based analyses were conducted for each animal or group to assess blood oxygenation level-dependent (BOLD) responses when the pad of the right hind limb was stimulated with von Frey filaments. The changes of BOLD signal intensity by the stimulation were calculated and indicated as SI change %.

### Statistical Analyses

All data are expressed as mean and SEM. The *n* values refer to the number of individual animals in each group on which experiments were performed. The statistical significance of differences was determined by using Wilcoxon rank sum test for two groups or Tukey's multiple comparison test with ANOVA or Steel-Dwass test for three groups. Two-way ANOVA was used for comparison of time course data. Probability values of <0.05 were considered significant. A test of no correlation was also performed. Statistical analyses were performed using JMP version 11.2.1 software (SAS Institute, Cary, NC, USA).

## Results

### Effects of Administration of Anti-IL-6 Receptor Antibody on Facial Grimacing in EAE Mice

**Figure 1:**
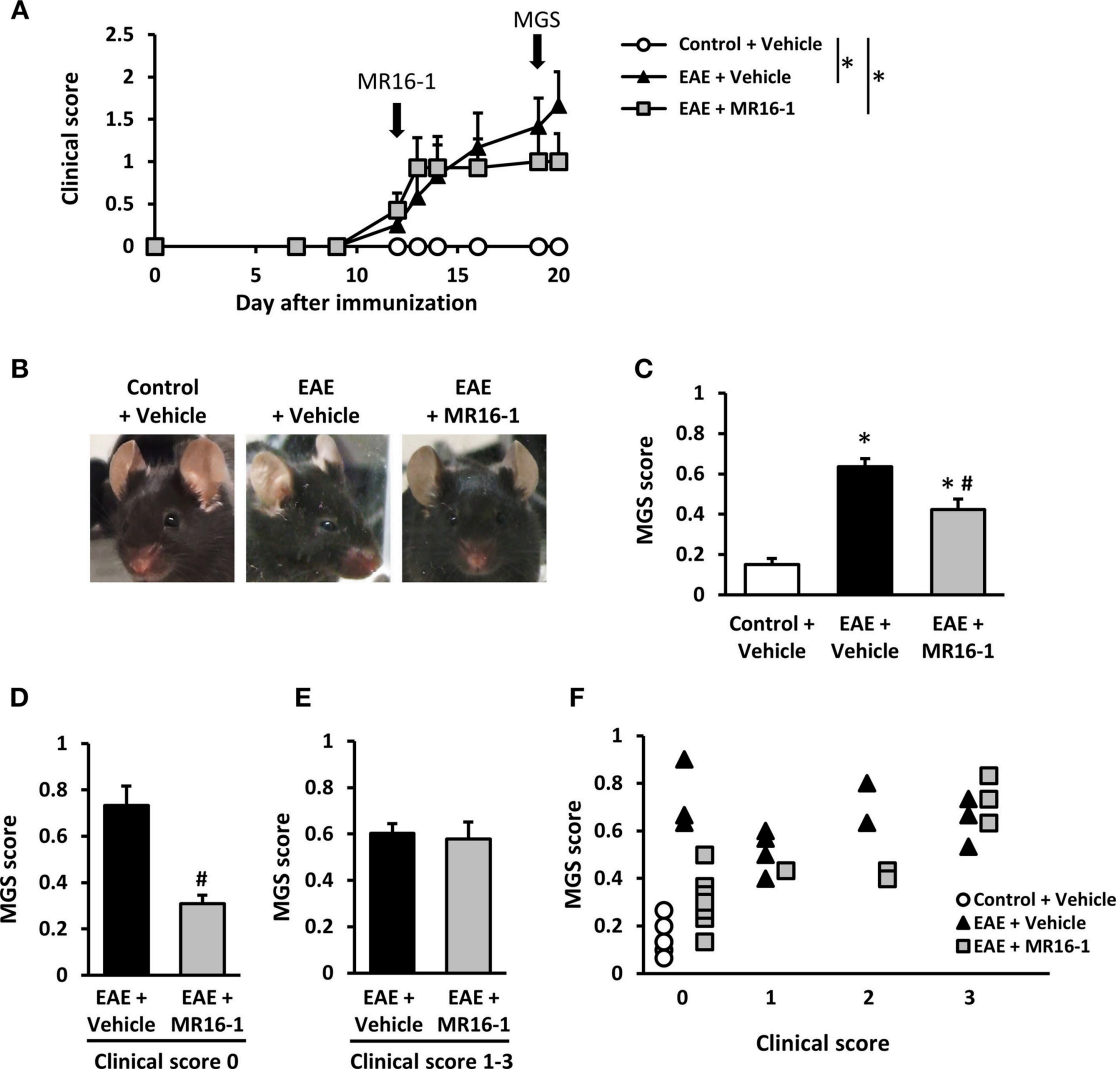
Effects of administration of MR16-1 on facial grimacing in EAE mice. **(A)** Administration of MR16-1 on Day 12 did not suppress clinical score in EAE mice. Arrows indicate the timing of MR16-1 administration and MGS measurement. **(B)** Representative images showing facial grimacing of control and EAE mice on Day 19 after immunization. **(C)** Administration of MR16-1 on Day 12 significantly decreased MGS score in EAE mice on Day 19. **(D–F)** Administration of MR16-1 significantly reduced MGS score in pre-onset EAE mice but not in post-onset EAE mice, especially in the mice with clinical score 3. ^*^p < 0.05 vs. Control + Vehicle, ^#^*p* < 0.05 vs. EAE + Vehicle by Steel-Dwass test or Wilcoxon rank sum test (*n* = 6–14 per group).

To evaluate spontaneous pain in EAE mice, we utilized the MGS. MGS score was significantly higher in EAE mice on Day 20 as compared to control mice in our preliminary study (Supplementary Figure S1). Administration of MR16-1 on Day 12 did not reduce clinical score in EAE mice (Figure 1A). This result corresponds to the previous study (12, 22). On the other hand, MR16-1 significantly decreased MGS score in EAE mice on Day 19 (Figures 1B,C). As far as the relationship between MGS score and clinical score could be confirmed, EAE mice treated with MR16-1 significantly reduced MGS score in pre-onset EAE mice (clinical score 0) (Figure 1D). On the other hand, we could not observe any clear effect of MR16-1 on MGS scores in post-onset EAE mice (clinical score 1–3), especially in the mice with clinical score 3 (Figures 1E,F).

### Effects of Administration of Anti-IL-6 Receptor Antibody on the Descending Pain Inhibitory System in Pre-onset EAE Mice

**Figure 2:**
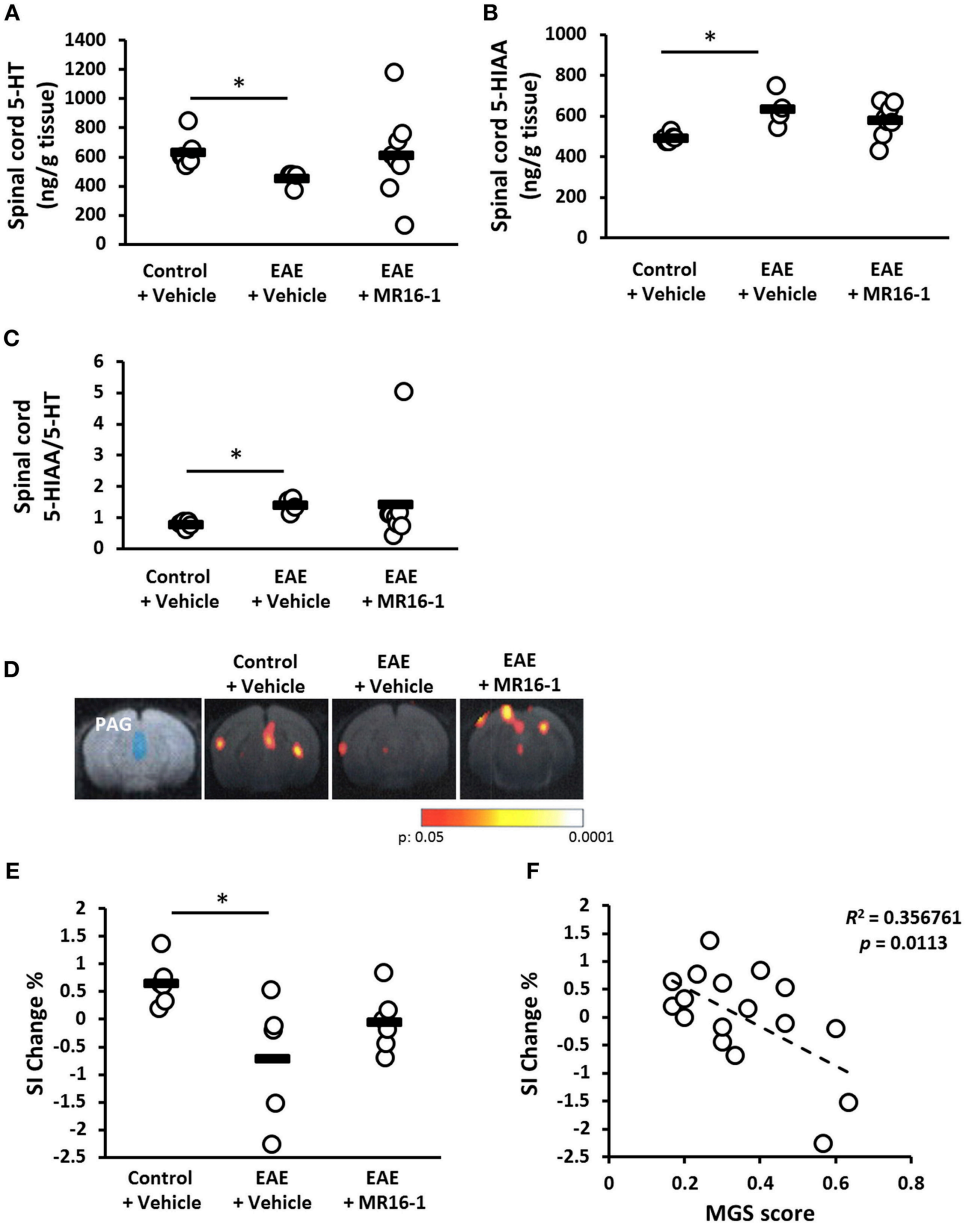
Effects of administration of MR16-1 on the descending pain inhibitory system in pre-onset EAE mice. **(A–D)** Spinal 5-HT and 5-HIAA was measured in control and pre-onset EAE mice (clinical score 0) on Day 20 after immunization. Levels of spinal 5-HT were decreased in EAE mice **(A)**. Levels of spinal 5-HIAA, the major metabolite of 5-HT, were increased in EAE mice **(B)**. 5-HIAA/5-HT ratio was increased in EAE mice **(C)**. ^*^p < 0.05 vs. Control + Vehicle by Steel-Dwass test (*n* = 4–8 per group). **(D)** Representative images of BOLD responses in the PAG. Blue area indicates PAG. Red and yellow areas show significant changes of BOLD signals. **(E)** The changes of BOLD signals in PAG were significantly decreased in pre-onset EAE mice. **(F)** There is a negative correlation between the changes of BOLD signal intensity in PAG and MGS score. Black bars indicate the mean value of each measurement. ^*^p < 0.05 vs. Control + Vehicle by Tukey's multiple comparison test (*n* = 5–6 per group).

Chronic pain conditions are frequently associated with a loss of endogenous inhibition systems, and serotonergic and noradrenergic projections to the spinal cord are important in descending pain modulation (25). Therefore, we measured the levels of spinal 5-HT and 5-HIAA in control and pre-onset EAE mice on Day 20. The levels of spinal 5-HT were decreased in vehicle-treated EAE mice (Figure 2A). The levels of 5-HIAA were increased in the spinal cord of vehicle-treated EAE mice (Figure 2B). Spinal cord 5-HIAA/5-HT ratio was increased in vehicle-treated EAE mice (Figure 2C), which means that spinal 5-HT turnover was promoted in EAE mice. Administration of MR16-1 showed higher mean level of spinal 5-HT and lower mean level of 5-HIAA in the spinal cord, but not significant (Figures 2A,B). Any differences by MR16-1 administration were not observed in the mean value of 5-HIAA/5-HT ratio in EAE mice (Figure 2C).

We also attempted to evaluate the neuronal activity in the periaqueductal gray (PAG), which modifies pain projecting neurons, i.e., serotonergic neurons, from the rostroventromedial medulla (RVM) to the spinal dorsal horn (26). In the present fMRI study, BOLD signal intensity in the PAG after the application of mechanical stimuli to the hind paw was significantly lower in pre-onset EAE mice than in control mice (Figures 2D,E). MR16-1 showed higher mean value of SI Change % compared with vehicle treatment, but not significant (Figure 2E). Checking the relationship between PAG function and MGS score revealed a negative correlation between BOLD signal intensity in the PAG and MGS score (Figure 2F).

## Discussion

This is the first report to indicate that anti-IL-6 receptor antibodies prevented spontaneous pain as evaluated by MGS score in EAE mice. In our study, MR16-1 clearly attenuated the MGS score in pre-onset EAE mice but not in post-onset EAE mice, especially in mice with a clinical score of 3. There was a tendency toward a negative correlation between body weight and MGS score in post-onset EAE mice but not in pre-onset EAE mice (Supplementary Figure S2). A previous report mentioned that it cannot be excluded that the MGS scores in post-onset EAE mice may be influenced by demyelination of lower motor neurons resulting in a compromised ability to exhibit facial expressions (27). Therefore, it may be possible that factors other than spontaneous pain were involved in increased MGS scores and that we could not correctly evaluate MGS as spontaneous pain in post-onset EAE mice. However, because the levels of spinal 5-HT and 5-HIAA/5-HT ratio were significantly improved by MR16-1 even in post-onset EAE mice (Supplementary Figure S3), MR16-1 might also have an effect on spontaneous pain in post-onset EAE mice. These results suggest that MR16-1 was effective on spontaneous pain in EAE mice, which correspond to the clinical reports indicated that IL-6 receptor antibodies ameliorated pain in NMO patients (28). Further examination is needed to clarify the details about the effect of anti-IL-6 receptor antibodies on spontaneous pain in post-onset EAE mice.

When pain signals reach the brain, the descending pain inhibitory system is mobilized from a variety of sites in the cerebral cortex and activates the PAG, which closely controls the RVM (26, 29). Serotonergic neurons in the RVM exert descending pain inhibition via projections to the spinal cord (26, 29, 30). Therefore, endogenous 5-HT in the spinal dorsal horn is known to greatly contribute to pain modulation (26, 31). Because 5-HT is re-uptaken or metabolized to 5-HIAA, the 5-HIAA/5-HT ratio reflects serotonergic activity (32). MR16-1 did not show significant changes in the levels of 5-HT and 5-HIAA/5-HT ratio in the spinal cord of pre-onset EAE mice due to its large variation (one mouse with a drastically low 5-HT and high 5-HIAA/5-HT ratio). In post-onset EAE mice, MR16-1 significantly increased 5-HT level and reduced 5-HIAA/5-HT ratio in the spinal cord (Supplementary Figure S3). Although further studies are required, we speculate that MR16-1 essentially modulates the serotonergic activity in the spinal cord of EAE mice.

We also attempted to evaluate neuronal activity in the PAG. BOLD signal intensity in the PAG after the application of mechanical stimuli to the hind paw was significantly lower in pre-onset EAE mice than in control mice. This result suggests that the descending pain inhibitory system was impaired in EAE mice. Furthermore, there was a negative correlation between BOLD signal intensity in the PAG and MGS score. A previous report also demonstrated that selective deficiencies in the descending inhibitory system resulted in elevated spontaneous neuron activity (33). Therefore, it seems that spontaneous pain was partly caused by impaired descending pain inhibitory system in EAE mice. In MS and NMO patients, periaqueductal lesions are typically observed (34, 35). Therefore, it is possible that one of the cause in the neuropathic pain in these patients is an impaired descending pain inhibitory system. Kawasaki et al. previously reported that IL-6 inhibited the frequency of spontaneous inhibitory postsynaptic currents in the spinal cord (36). In this study, although MR16-1 did not significantly inhibited the reduction of BOLD signal intensity in the PAG of pre-onset EAE mice due to its large variation, we anticipate that MR16-1 has some potentials to prevent PAG dysfunction. Further studies are needed to clarify the effect of MR16-1 on PAG dysfunction in EAE mice.

In addition to spinal 5-HT, noradrenergic projections to the spinal dorsal horn are also important in descending pain modulation. A previous study demonstrated that the analgesic effects of pregabalin are induced via the descending noradrenergic system, especially the increase of 3-methoxy-4-hydroxyphenylglycol (MHPG, metabolite of noradrenaline) in the spinal cord (37). In our study, we also tried to detect the spinal MHPG, but unfortunately we could not measure MHPG in EAE mice. Therefore, we cannot exclude the possibility that noradrenaline may be important in the spontaneous pain in EAE mice, and further examinations are needed to clarify the details.

In summary, MR16-1 prevented spontaneous pain in EAE mice, especially in pre-onset phase. This spontaneous pain was partly caused by impaired descending pain inhibitory system. As the limitation of this study, MR16-1 did not show any significant changes in descending pain inhibitory systems due to its large variation. Further studies using more number of animals are required to clarify the detailed mechanisms of the analgesic effects of MR16-1.

## Ethics Statement

All experimental protocols were approved by the Animal Care Committee of the institution and conformed to the Guide for the Care and Use of Laboratory Animals published by the US National Institutes of Health.

## Author Contributions

KS: conception, design, performing experiments, data acquisition, and drafting of the article. HT-S: design, performing experiments, and data acquisition. HY: performing experiment. KY: conception, and design. YM: responsibility for the integrity of the study. All authors have reviewed the article and have approved the final manuscript.

### Conflict of Interest Statement

All authors are employees of Chugai Pharmaceutical Co., Ltd.

## References

[1] Kanamori Y Nakashima I Takai Y Nishiyama S Kuroda H Takahashi T. Pain in neuromyelitis optica and its effect on quality of life: a cross-sectional study. Neurology. (2011) 77:652–8. 10.1212/WNL.0b013e318229e694 21813781

[2] Osterberg A Boivie J Thuomas KA. Central pain in multiple sclerosis–prevalence and clinical characteristics. Eur J Pain. (2005) 9:531–42. 10.1016/j.ejpain.2004.11.005 16139182

[3] Zhao S Mutch K Elsone L Nurmikko T Jacob A. Neuropathic pain in neuromyelitis optica affects activities of daily living and quality of life. Mult Scler. (2014) 20:1658–61. 10.1177/1352458514522103 24493470

[4] Kessler RA Mealy MA Levy M. Treatment of neuromyelitis optica spectrum disorder: acute, preventive, and symptomatic. Curr Treat Options Neurol. (2016) 18:2. 10.1007/s11940-015-0387-9 26705758 PMC4807395

[5] Svendsen KB Jensen TS Hansen HJ Bach FW. Sensory function and quality of life in patients with multiple sclerosis and pain. Pain. (2005) 114, 473–81. 10.1016/j.pain.2005.01.015 15777872

[6] Zhou YQ Liu Z Liu ZH Chen SP Li M Shahveranov A. Interleukin-6: an emerging regulator of pathological pain. J Neuroinflammation. (2016) 13:141. 10.1186/s12974-016-0607-6 27267059 PMC4897919

[7] DeLeo JA Colburn RW Nichols M Malhotra A. Interleukin-6-mediated hyperalgesia/allodynia and increased spinal IL-6 expression in a rat mononeuropathy model. J Interferon Cytokine Res. (1996) 16:695–700. 10.1089/jir.1996.16.695 8887053

[8] Ramer MS Murphy PG Richardson PM Bisby MA. Spinal nerve lesion-induced mechanoallodynia and adrenergic sprouting in sensory ganglia are attenuated in interleukin-6 knockout mice. Pain. (1998) 78:115–21. 10.1016/S0304-3959(98)00121-3 9839821

[9] Murphy PG Ramer MS Borthwick L Gauldie J Richardson PM Bisby MA. Endogenous interleukin-6 contributes to hypersensitivity to cutaneous stimuli and changes in neuropeptides associated with chronic nerve constriction in mice. Eur J Neurosci. (1999) 11:2243–53. 10.1046/j.1460-9568.1999.00641.x 10383613

[10] Arruda JL Sweitzer S Rutkowski MD DeLeo JA. Intrathecal anti-IL-6 antibody and IgG attenuates peripheral nerve injury-induced mechanical allodynia in the rat: possible immune modulation in neuropathic pain. Brain Res. (2000) 879:216–25. 10.1016/S0006-8993(00)02807-9 11011025

[11] Murakami T Kanchiku T Suzuki H Imajo Y Yoshida Y Nomura H. Anti-interleukin-6 receptor antibody reduces neuropathic pain following spinal cord injury in mice. Exp Ther Med. (2013) 6:1194–8. 10.3892/etm.2013.1296 24223643 PMC3820708

[12] Serizawa K Tomizawa-Shinohara H Magi M Yogo K Matsumoto Y. Anti-IL-6 receptor antibody improves pain symptoms in mice with experimental autoimmune encephalomyelitis. J Neuroimmunol. (2018) 319:71–9. 10.1016/j.jneuroim.2018.03.017 29685293

[13] Baxter AG. The origin and application of experimental autoimmune encephalomyelitis. Nat Rev Immunol. (2007) 7:904–12. 10.1038/nri2190 17917672

[14] Dutra RC Bento AF Leite DF Manjavachi MN Marcon R Bicca MA. The role of kinin B1 and B2 receptors in the persistent pain induced by experimental autoimmune encephalomyelitis (EAE) in mice: evidence for the involvement of astrocytes. Neurobiol Dis. (2013) 54:82–93. 10.1016/j.nbd.2013.02.007 23454198

[15] Olechowski CJ Tenorio G Sauve Y Kerr BJ. Changes in nociceptive sensitivity and object recognition in experimental autoimmune encephalomyelitis (EAE). Exp Neurol. (2013) 241:113–21. 10.1016/j.expneurol.2012.12.012 23291347

[16] Olechowski CJ Truong JJ Kerr BJ. Neuropathic pain behaviours in a chronic-relapsing model of experimental autoimmune encephalomyelitis (EAE). Pain. (2009) 141:156–64. 10.1016/j.pain.2008.11.002 19084337

[17] Truini A Garcia-Larrea L Cruccu G. Reappraising neuropathic pain in humans–how symptoms help disclose mechanisms. Nat Rev Neurol. (2013) 9:572–82. 10.1038/nrneurol.2013.180 24018479

[18] Backonja MM Stacey B. Neuropathic pain symptoms relative to overall pain rating. J Pain. (2004) 5:491–7. 10.1016/j.jpain.2004.09.001 15556827

[19] Mogil JS. The etiology and symptomatology of spontaneous pain. J Pain. (2012) 13:932–33; discussion 934–5. 10.1016/j.jpain.2012.07.006 23031393

[20] Murai N Sekizawa T Gotoh T Watabiki T Takahashi M Kakimoto S. Spontaneous and evoked pain-associated behaviors in a rat model of neuropathic pain respond differently to drugs with different mechanisms of action. Pharmacol Biochem Behav. (2016) 141:10–7. 10.1016/j.pbb.2015.11.008 26597514

[21] Okazaki M Yamada Y Nishimoto N Yoshizaki K Mihara M. Characterization of anti-mouse interleukin-6 receptor antibody. Immunol Lett. (2002) 84:231–40. 10.1016/S0165-2478(02)00202-X 12413742

[22] Serada S Fujimoto M Mihara M Koike N Ohsugi Y Nomura S. IL-6 blockade inhibits the induction of myelin antigen-specific Th17 cells and Th1 cells in experimental autoimmune encephalomyelitis. Proc Natl Acad Sci USA. (2008) 105:9041–6. 10.1073/pnas.0802218105 18577591 PMC2449361

[23] Langford DJ Bailey AL Chanda ML Clarke SE Drummond TE Echols S. Coding of facial expressions of pain in the laboratory mouse. Nat Methods. (2010) 7:447–9. 10.1038/nmeth.1455 20453868

[24] Leite FP Mandeville JB. Characterization of event-related designs using BOLD and IRON fMRI. Neuroimage. (2006) 29:901–9. 10.1016/j.neuroimage.2005.08.022 16213164

[25] Ossipov MH Morimura K Porreca F. Descending pain modulation and chronification of pain. Curr Opin Support Palliat Care. (2014) 8:143–51. 10.1097/SPC.0000000000000055 24752199 PMC4301419

[26] Millan MJ. Descending control of pain. Prog Neurobiol. (2002) 66:355–474. 10.1016/S0301-0082(02)00009-6 12034378

[27] Duffy SS Perera CJ Makker PG Lees JG Carrive P Moalem-Taylor G. Peripheral and central neuroinflammatory changes and pain behaviors in an animal model of multiple sclerosis. Front Immunol. (2016) 7:369. 10.3389/fimmu.2016.00369 27713744 PMC5031691

[28] Araki M Matsuoka T Miyamoto K Kusunoki S Okamoto T Murata M. Efficacy of the anti-IL-6 receptor antibody tocilizumab in neuromyelitis optica: a pilot study. Neurology. (2014) 82:1302–6. 10.1212/WNL.0000000000000317 24634453 PMC4001188

[29] Bliss TV Collingridge GL Kaang BK Zhuo M. Synaptic plasticity in the anterior cingulate cortex in acute and chronic pain. Nat Rev Neurosci. (2016) 17:485–96. 10.1038/nrn.2016.68 27307118

[30] Fields HL Heinricher MM Mason P. Neurotransmitters in nociceptive modulatory circuits. Annu Rev Neurosci. (1991) 14:219–45. 10.1146/annurev.ne.14.030191.001251 1674413

[31] Obata H. Analgesic mechanisms of antidepressants for neuropathic pain. Int J Mol Sci. (2017) 18:E2483. 10.3390/ijms18112483 29160850 PMC5713449

[32] Shannon NJ Gunnet JW Moore KE. A comparison of biochemical indices of 5-hydroxytryptaminergic neuronal activity following electrical stimulation of the dorsal raphe nucleus. J Neurochem. (1986) 47:958–65. 10.1111/j.1471-4159.1986.tb00704.x 2426412

[33] Patel R Qu C Xie JY Porreca F Dickenson AH. Selective deficiencies in descending inhibitory modulation in neuropathic rats: implications for enhancing noradrenergic tone. Pain. (2018) 159:1887–99. 10.1097/j.pain.0000000000001300 29863529 PMC6095727

[34] Papadopoulou A Naegelin Y Weier K Amann M Hirsch J von Felten S. MRI characteristics of periaqueductal lesions in multiple sclerosis. Mult Scler Relat Disord. (2014) 3:542–51. 10.1016/j.msard.2014.01.001 25877067

[35] Tackley G Kuker W Palace J. Magnetic resonance imaging in neuromyelitis optica. Mult Scler. (2014) 20:1153–64. 10.1177/1352458514531087 24829291

[36] Kawasaki Y Zhang L Cheng JK Ji RR. Cytokine mechanisms of central sensitization: distinct and overlapping role of interleukin-1beta, interleukin-6, and tumor necrosis factor-alpha in regulating synaptic and neuronal activity in the superficial spinal cord. J Neurosci. (2008) 28:5189–94. 10.1523/JNEUROSCI.3338-07.2008 18480275 PMC2408767

[37] Takeuchi Y Takasu K Ono H Tanabe M. Pregabalin, S-(+)-3-isobutylgaba, activates the descending noradrenergic system to alleviate neuropathic pain in the mouse partial sciatic nerve ligation model. Neuropharmacology. (2007) 53:842–53. 10.1016/j.neuropharm.2007.08.013 17889907

