# Supplementary material for: Anti-IL-6 Receptor Antibody Inhibits Spontaneous Pain at the Pre-onset of Experimental Autoimmune Encephalomyelitis in Mice
Source: Front Neurol. 2019 Apr 9;10:341. doi: 10.3389/fneur.2019.00341 (PMC6465542; doi:10.3389/fneur.2019.00341)
Supplement: Supplementary file 1 [file Data_Sheet_1.PDF]

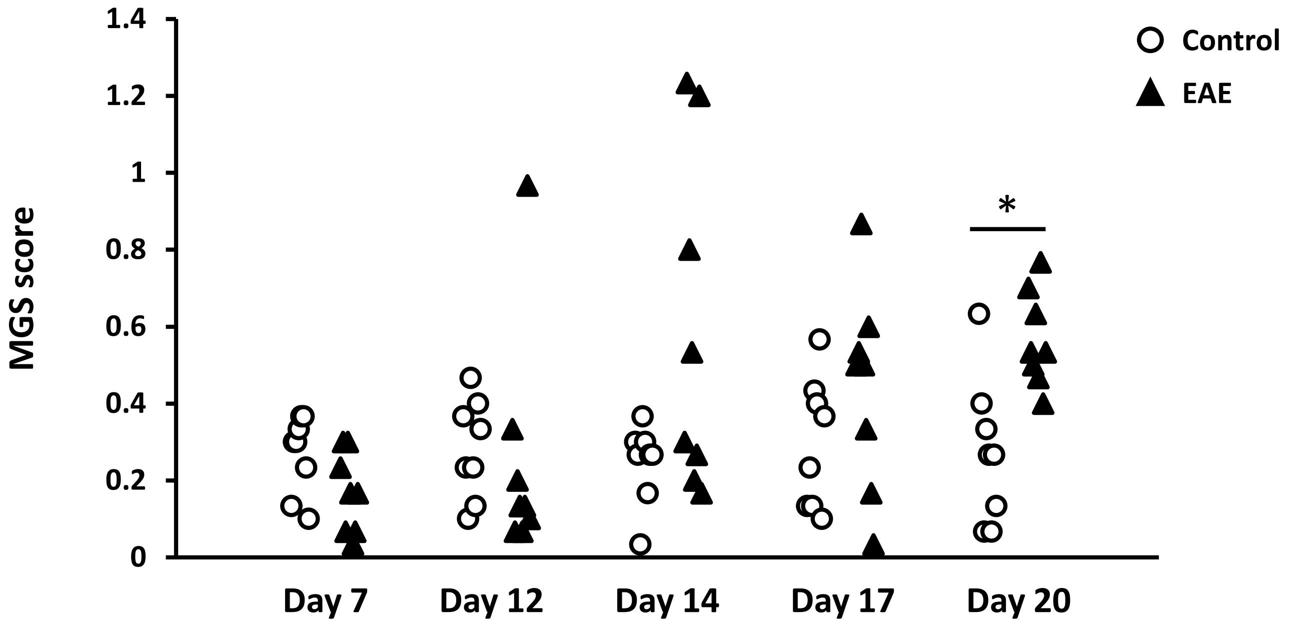

Supplementary Fig. S1. Evaluation of facial grimacing in EAE mice.
MGS score was significantly increased in EAE mice at Day 20 after immunization as compared to control mice. *$p < 0.05$ *vs.* Control by Wilcoxon rank sum test ($n = 8$ per group).

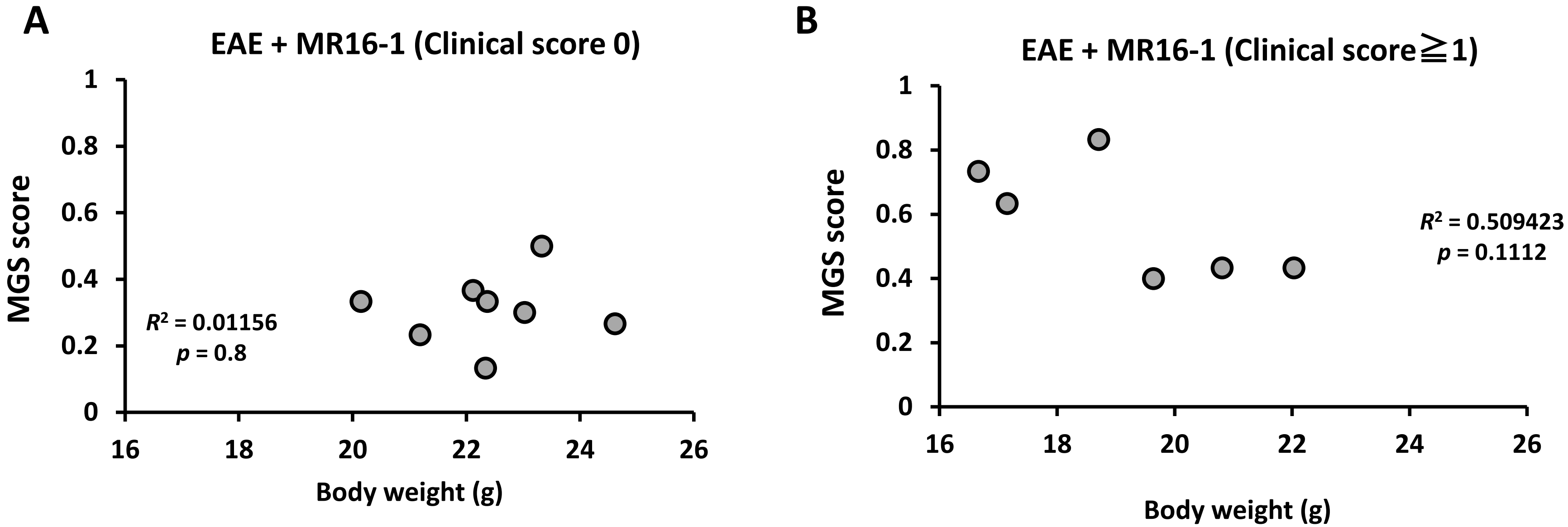

Supplementary Fig. S2. Relationship between MGS score and body weight in pre- and post-onset EAE mice treated with MR16-1.
(A) There is no correlation between MGS score and body weight in pre-onset EAE mice treated with MR16-1. **(B)** MGS score showed a tendency towards a negative correlation with body weight in post-onset EAE mice treated with MR16-1. MGS and body weight were measured at Day 19 after immunization.

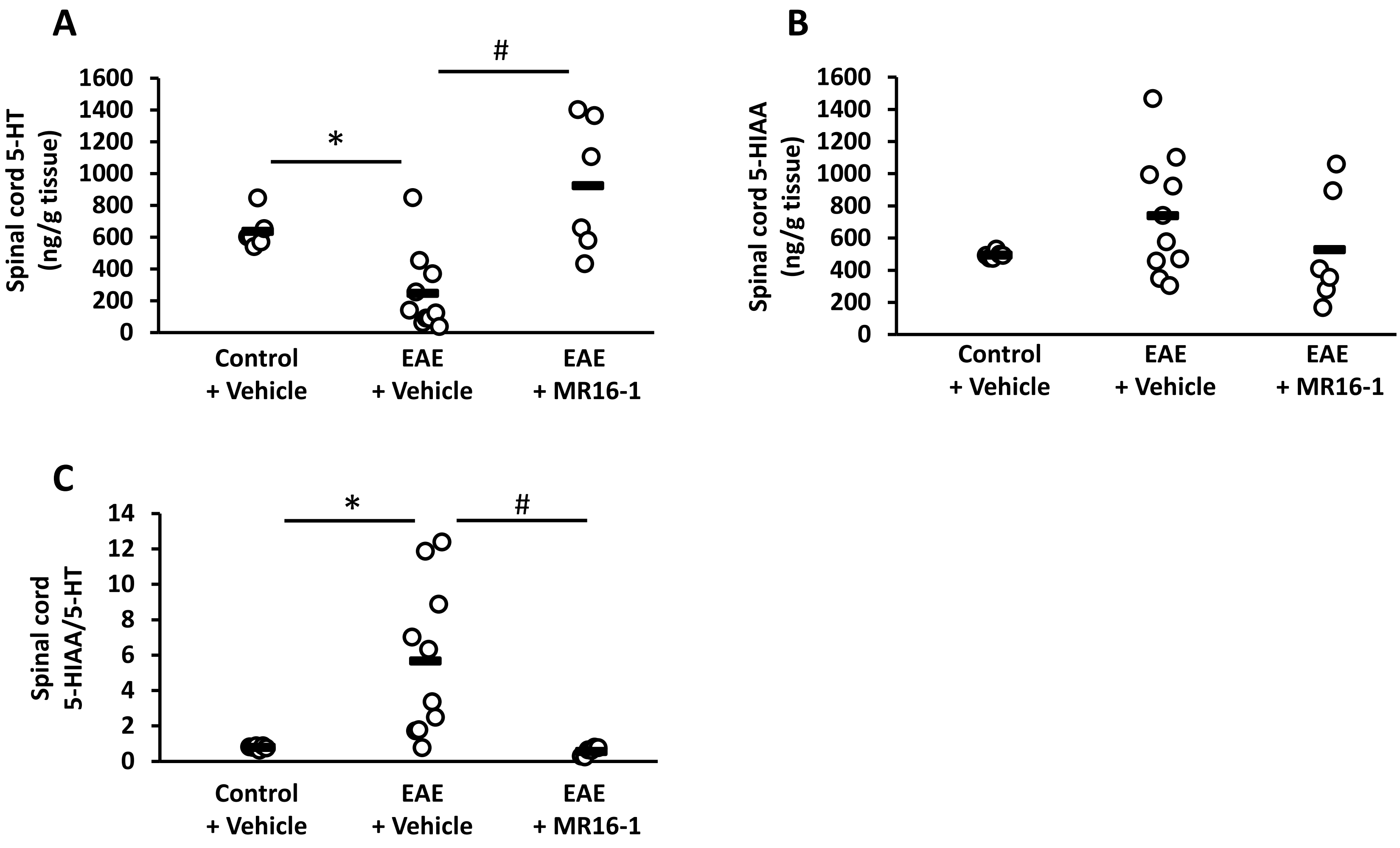

Supplementary Fig. S3. Effects of administration of MR16-1 on spinal 5-HT in post-onset EAE mice.
(A–C) Spinal 5-HT and 5-HIAA were measured in control and post-onset EAE mice (clinical score 1-4) at Day 20 after immunization. Levels of spinal 5-HT were decreased in EAE mice and MR16-1 prevented it **(A)**. Levels of spinal 5-HIAA were not changed between the groups **(B)**. 5-HIAA/5-HT ratio was increased in EAE mice and MR16-1 inhibited it **(C)**. $^{*}p < 0.05$ *vs.* Control + Vehicle, $^{\#}p < 0.05$ *vs.* EAE + Vehicle by Steel-Dwass test (n = 6–10 per group).
